# Video Analysis of Parent–Child Interactions in Behavioral Sleep Disorders: Development of a Scoring Algorithm

**DOI:** 10.3389/fpsyt.2019.00861

**Published:** 2019-11-22

**Authors:** Lorna Galbraith, Kim Bull, Catherine M. Hill

**Affiliations:** ^1^Faculty of Medicine, University of Southampton, Southampton, United Kingdom; ^2^Children’s Sleep Disorder Service, Southampton Children’s Hospital, Southampton, United Kingdom

**Keywords:** sleep, tool, child, insomnia, behavior

## Abstract

**Introduction:** Behavioral sleep disorders, including chronic insomnia (CI), are generally assessed by subjective parent interview. However, evidence suggests that parental report of children’s overnight behaviors is unreliable, perhaps due to recall bias or confusion due to sleep deprivation. Video technology has been used clinically to capture complex behavioral disorders in children during the day. However, there is no standardized means of analyzing child and parent behavior at bedtime or during the night. We aimed to create an algorithm for this purpose.

**Methods:** Child brain tumor survivors (a population previously shown to have a high prevalence of CI) were screened for difficulties initiating and maintaining sleep using sub-scales from the Sleep Disturbance Scale for Children. Those who screened positive (n = 3) then completed a detailed parent interview to confirm a clinical diagnosis of CI. One night of home video footage was obtained from initial settling period to morning waking (SOMNOmedics camera). Footage was imported into BORIS^©^ software and a coding system for parent and child behavior was developed over multiple iterations until agreeable inter-rater reliability (>70%) was achieved between two independent coders.

**Results:** The final coding categories were: 1) time domains, 2) physical environment, 3) child global status, 4) location, 5) activity, and 6) physical interaction. This achieved 74% inter-reliability in its last iteration.

**Discussion:** A statistically acceptable behavior scoring algorithm was achieved. With further development, this tool could be applied clinically to investigate behavioral insomnia and in research to provide more objective outcome measurement.

## Introduction

Chronic insomnia (CI) is the commonest childhood sleep disorder (1). According to the Third Edition of the International Classification of Sleep Disorders (ICSD-3), a diagnosis of CI requires 1) difficulty initiating and/or maintaining sleep, 2) adequate opportunity to do so, and 3) subsequent daytime dysfunction. The symptoms must occur at least three times per week and have been present for at least 3 months (2). CI leads to insufficient sleep which can be detrimental to a child’s behaviour (3), cognitive function (4), and brain development (5).

The commonest CI in childhood is behavioral insomnia. There are two core sub-types.

1. Sleep onset association disorder, where the child requires specific “associations,” for example a parent/carer present in the room or environmental factors such as music or light, to fall asleep. (From here on “parent” will refer to any carer with involvement in the bedtime routine). With these associations, the child settles to sleep easily but following natural night wakings struggles to resettle to sleep without the same conditions they have associated with bedtime. Typically, the child then signals for a parent by crying or leaving the room (6), this is interpreted as a troublesome night waking.
2. Limit setting sub-type, where the child makes repeated requests to the parent in an attempt to delay his/her bedtime, that is, “curtain calls”. This is accommodated by the parent, who fails to enforce sufficient boundaries (6).

Where elements of both sleep onset association insomnia and limit setting insomnia are present, the term “combined type insomnia” (6) is used.

The standard diagnostic assessment of CI is through a clinical history, which has some limitations. First, while parental recall of their child’s *sleep schedule* (i.e., sleep onset and duration) has been shown to correlate with objective measures (actigraphy), overnight *sleep quality* [the child’s overnight activity including number of night wakings (7)] is less reliably reported (8, 9). Parents are only able to report activity of which they are aware. If the child does not disturb the parent, these wakings may be overlooked9. While the child’s own account is relevant, many children with CI may not have the developmental skills to provide an accurate history. Furthermore, parental recall may be restricted by their own memory of events, which may be impaired when tired, particularly after repeated awakenings (9). On occasions the sleep problems may be exaggerated, thus misleading any intervention put in place by the clinician (10).

An important etiological factor in the development of CI is parent behavior. This is usually communicated by self-report so is dependent on the parent’s awareness of his or her own actions. Additionally, parents have been shown to sometimes be untruthful when asked to give a self-report of their parenting due to social desirability bias. For example, they may tell clinicians that their child is up-to-date with their vaccines when they are not (11). An objective measure of both child sleep and parent-child interaction would therefore be of value.

Actigraphy is the commonest objective measure of sleep in the child’s home environment. It detects overnight activity by measuring limb movement *via* a wrist-worn device (12). However, it does not detect wakeful stillness, therefore its validity as an objective measure in insomnia patients is limited (13).

Direct observation of parent behavior is more accurate than parent self-report (14, 15). However, the presence of an observer influences both child and parent behavior, and it is costly and impractical in most clinical settings. Home video somnography (HVSG) is a simple technique using an infra-red camera setup in the child’s bedroom that measures sleep and captures information about the child’s usual sleep ecology (sleep setting and parent behaviors) without the need for body worn sensors. HVSG was pioneered in the 1970s by Anders and colleagues (16–18) to assess developmental changes in infant sleep. In these original studies basic sleep states were scored (awake, active sleep, quiet sleep). VSG has been since used across multiple applications, principally to assess episodic movement disorders (19) in sleep. The advent of smartphone cameras and low cost motion sensitive night cameras has brought this application into every household, and increasingly, parents and clinicians are exploiting this technology in the clinic to report sleep-related symptoms (20).

However, the use of HVSG for behavioral analysis at bedtime and during the night is new. Only one case study has been published to date using HVSG to observe and code parent and child behavior in CI (21). The authors used a basic coding system to analyze the child’s sleep quantitatively. Only one coder assessed the footage, therefore no inter-rater reliability was calculated. There are a number of established coding systems for daytime parent-child interactions (22–24) designed for specific situations, such as to measure infant soothing and distress (22), or to code parent and child interactions over a short duration, such as the Iowa Family Interaction Rating Scale (23). A recent study investigated the scoring of motion detection video recording to measure sleep disturbance in three children aged 3 to 5 years with autism spectrum disorder (25). This study found that there was 100% agreement between two observers who scored night wakings and sleep disturbance (within a five second interval of each other) using the video footage. The authors acknowledged that this technology could be applied to monitor parent-child interactions which might contribute to problematic sleep behavior.

We have previously reported that sleep problems are prevalent in survivors of childhood brain tumors (26). In a clinical population of brain tumor survivors, 37/55 (67%) of participants aged 3 to 27 years were at risk of having a sleep disorder based on validated questionnaires (Children’s Sleep Habits Questionnaire, Pittsburgh Sleep Quality Index, and the Epworth Sleepiness Scale). The study indicated that the commonest sleep disorder experienced in this population was likely to be CI, independent of both the type of treatment received and the tumor’s location in the brain. We therefore hypothesized that CI in brain tumor survivors had a behavioral etiology. However after some preliminary research, we found that there was no standardized method to analyze sleep-related behavior. Therefore, for this study, we selected childhood brain tumor survivors, a population we knew were likely to have a high proportion of behavioral sleep disorders, to produce and test a tool to analyze sleep behaviors.

We aimed: a) to develop a reliable (i.e. reproducible), practical parent-child behavior scoring tool for use with an overnight home video recording of the child’s bedtime and overnight activity and b) to compare parent-report of child sleep with HVSG objective measures.

## Methods

Ethical approval was obtained from the Health Research Authority and South Central—Hampshire A Research Ethics Committee and research governance approval from University Hospital Southampton NHS Foundation Trust.

### Materials

Children were screened for eligibility using the Disorders of Initiating and Maintaining Sleep subscale of the Sleep Disturbance Scale for Children (SDSC). This consists of 7 questions rated on a 5-point Likert scale pertaining to the previous 6 months with a maximum score of 35. If the child scored 16 or more they were eligible for further assessment.

Equipment used for HVSG was the SOMNOmedics infrared camera with SOMNOwatch actigraphy. This is a portable camera designed to capture activity in low light conditions along with audio recording. The data are downloaded to DOMINO Light^™^ software that synchronizes actigraphy data with video footage, allowing the coder to rapidly locate periods of movement. However, this software only allows basic marking of the video in real time and had limitations for the analysis of multi-layered aspects of sleep ecology.

An alternative software, BORIS^©^ (Behavior Observation Research Interactive Software), was identified. This was developed by researchers at the University of Turin to document animal behavior. BORIS^©^ offers the requisite features for this study, namely, it was intuitive to use and allowed the coder to classify behaviors as *state* events, which are ongoing (e.g. when the child is asleep), or *point* events which happen at a single point in time (e.g. time at sleep onset).

### Data Source

Eligible potential children (aged 3 to 12 years who had completed brain tumor treatment at least six months previously) were pre-screened for CI using the SDSC questionnaire. Three children who were at risk of CI were recruited.

Recruited children underwent one night of HVSG at home. Recording was programmed to begin prior to the start of their normal bedtime routine, and the parent was instructed to stop the recording when the child woke up in the morning. Families were instructed to carry out their normal bedtime routine, therefore the camera captured all interactions happening in the bedroom during the bedtime routine and during the night.

The day following the overnight recording, parents were interviewed by the researcher LG (Lorna Galbraith) using a structured clinical interview (Southampton Children’s Hospital Sleep Service). This interview was audio-recorded, and the recording was used to confirm or reject a clinical diagnosis of CI and to obtain the caregiver’s self-report of overnight activity.

### Coding System Development

Crano’s principles of coding system development were employed (27). This specifies that the following options are considered: a) a categorical or a rating system (in this case a categorical system was more appropriate to incorporate a wide range of behaviors), b) an intensive or extensive system (an intensive approach was initially selected to analyze interactions in detail), and c) a non-inferential or inferential system, the latter meaning that the coder must consider the function of a person’s action as well as the action itself (27). A partially inferential approach was adopted, to reflect the fact that the emotional tone of any behavior was important to the outcome (sleep onset) rather than the action itself. For example, a parent can soothe their child during the bedtime routine by singing a lullaby gently; however, if the singing is loud and stimulating, this will not help to settle the child. The authors applied their expertise in sleep medicine (Dr Catherine Hill - CH) and psychology of childhood brain tumor survivors (Dr Kim Bull [KB]) to the development of the parent-child interaction coding system. A draft version of a coding algorithm was developed using the following headings: 1) sleep schedule variables, 2) events during the bedtime routine, 3) events after lights out, 4) events after a night waking, and 5) parent-child interactions. Parent-child interactions were further dichotomized as either sleep-promoting or sleep-hindering, as determined by the coder. Physical and verbal behaviors were separated. The full list of codes from this first draft of the coding system can be seen in Table 1.

**Table 1:** Initial draft of the Overnight Parent-Child Interaction Coding System. The first iteration of the coding system, constructed by the research team based on the clinical expertise of an experienced pediatric sleep consultant.

Category	Event
Sleep schedule	Bedtime routine (S)
	Lights out (P)
	Sleep (P)
	Night waking (P)
	Wake time (P)
	Physical contact brief (P)
	Parent present not engaging (S)
	Sleep onset (P)
Bedtime routine	Reading (S)
	Singing (S)
	Playing (S)
	Electronics (S)
	Physical contact extended (S)
After lights out	Self soothes (P)
	Signals (P)
	Activity (S)
	Leaves room (P)
Night waking	Self soothes (P)
	Signals (P)
	Activity (S)
	Leaves room (P)
	Drinks (S)
	Movement arousal (S)
Parent positive commentary	Positive statement (P)
	Offers reward (P)
	Praise (P)
	Reassure (P)
	Calm response (P)
	Authoritative statement (P)
Parent negative commentary	Raises voice (P)
Parent positive behavior	Authoritative action (P)
	No response (P)
	Parent ignoring child (P)
Parent negative behavior	Fulfills curtain call (P)
	Parent engaging w/child (S)
	Soothing behavior (S)
Child positive commentary	Child agrees (P)
Child negative commentary	Settling environment (P)
	Settling process (P)
	Anxious comment (P)
Disagrees (P)	
Child positive behavior	Positive behavior change (P)
Child negative behavior	Non-compliant behavior (S)
	Physical resistance (P)
	Tantrum (S)
	Curtain call (P)

P, point event; S, state event. A point event is a brief event occurring in a single moment; a state event is a prolonged behavior with a marked start and end point.

The coding system was then tested using the video data from the overnight footage. LG coded 10-min segments of video data with high levels of parent-child interaction using BORIS^©^. CH independently coded these segments. Any disagreements were discussed, and the coding system was modified accordingly. During this process a coding system manual was constructed. This iterative process was repeated multiple times until all observed behaviors were accounted for and defined in a coding manual.

### Reliability of the Coding System

The coding system was then tested for inter-rater reliability (IRR). LG and CH each independently coded a 20-min segment of video during a bedtime routine. A modified percentage agreement was used to calculate IRR by adding up the number of codes with exact agreement between the two coders, within a time frame margin of five seconds, and dividing by the total number of codes. The percentage agreement statistic was selected since it is straight-forward to calculate, given the relative complexity of the multi-layer coding system, and is intuitive to interpret; 0% agreement means complete disagreement, whilst 100% means total agreement. An inter-rater percentage agreement of 70% has been reported to be sufficient for complex systems (28). Alternative statistics for IRR were considered, such as Cohen’s kappa, but this can be easily skewed if certain behaviors are more frequently coded than others (29), which we predicted would be the case. Additionally, Cohen’s kappa is used to exclude agreement which would have occurred by chance—since the draft coding system was relatively complex, with 46 different behaviors, chance agreement was deemed unlikely. Percentage agreement was therefore calculated for the coding system.

### Comparison of VSG Data to Parental Report

Once reliability of the coding system was considered to be acceptable, LG coded the video footage in full using BORIS^©^, including the bedtime routines and any night wakings. The coded data were compared to parental interview to assess the accuracy of parental self-report of the child’s sleep schedule (i.e. time at sleep onset and morning waking) and the child’s sleep quality (i.e. number of night wakings and night waking duration).

Following the analysis of the video, each participating family was contacted to discuss any findings of the overnight study, and if the child was found to have a significant sleep problem, the family were offered an appointment with the Southampton Children’s Hospital Sleep Service.

## Results

### Participants

Three children aged between 3 and 8 years, who were deemed to be at risk of CI following screening, were recruited. Demographic data can be seen in Table 2, though this has been abridged to protect participant anonymity. Parental interview confirmed a clinical diagnosis of CI in participant 1, however participants 2 and 3 did not meet the diagnostic criteria for CI.

**Table 2:** – Participant demographics.

Participant number	Age (years)	Years since end of treatment	Co-morbidities	SDSC**2** screening score (/35)
**1**	8.28	6.43	Hydrocephalus, hormone deficiency, epilepsy, developmental delay	24
**2**	7.49	1.94	Hormone deficiency	16
**3**	3.94	2.21	Hydrocephalus, hormone deficiency, facial palsy, hearing loss, developmental delay	18

SDSC, Sleep Disturbance Scale for Children. A score of 16 or more indicates an increased risk of a Disorder of Initiating or Maintaining Sleep. All children were above this threshold.

### The Overnight Parent-Child Interaction Coding System

Thirty-six hours of overnight footage were produced during data collection, of which nearly 3 h involved parent-child interaction. These video data were used to develop and test the reliability of the coding system.

The final coding system is depicted in Table 3. It comprises twenty-six codes divided into six categories. These categories were as follows:

**Table 3:** Final version of the Overnight Parent-Child Interaction Coding System. The final iteration of the coding system used to analyze parent and child behaviors at bedtime and during the night.

Category	Event	Modifier
Time domains	Settling routine (S)	
	“Lights out” (P)	
	Sleep onset (P)	
	Morning waking (P)	
Physical environment	Noise intrusion (S)	
	Music (S)	
	Bright lighting (S)	
Child global state	Sleep (S)	
	Night waking (S)	
	Movement arousal (S)	
Location	In bed (S)	*Position:*
		Lying Sitting Standing On all fours Crouching Mobile
Out of bed (S)		
Activity	Laughing (P)	*Helpful to sleep?*
		Soothing Non-soothing Neutral
	Reading (S)	
	Singing (S)	
	Playing (S)	
	Eating (S)	
	Drinking (S)	
	Verbalization (S)	
	Rocking (S)	
	Tidying/housekeeping (S)	
Personal care (S)		
	Electronics (S)	
	Self-stimulating (S)	
Physical interaction	Brief close physical contact (P)	
	Extended close physical contact (S)	

P, point event, S, state event. A point event is a brief event occurring in a single moment; a state event is a prolonged behavior with a marked start and end point.

1. *Time domains.* This allows the observer to record “sleep schedule” variables of the child’s sleep, i.e., sleep onset and morning waking, as well as the timing of the settling routine and the time at “lights out” (i.e. when the settling routine finishes).
2. *Physical environment.* The observer can record changes in the bedroom environment, such as loud sounds or changes in light which might be disruptive to the settling process.
3. *Child global status.* These codes relate to the child’s sleep/wake status, including any night wakings.
4. *Location.* The observer can record whether the subject (parent or child) is in or out of the bed and their position (e.g. sitting, lying). This was included because frequent changes of the child’s position may indicate that the bedtime routine is not having the desired settling effect. Additionally, presence of the parent in the room when the child falls asleep may be indicative of a sleep onset association.
5. *Activity*. The observer can record various activities occurring in the bedroom (e.g. reading, playing a game, singing), and indicate whether the behavior is “soothing” or “non-soothing” based on the child’s reaction to the activity (i.e. whether they settle or become excited/agitated).
6. *Physical interaction*. The observers can record either brief or extended close physical contact. This is significant as physical contact could be a beneficial soothing aspect of a bedtime routine, however it could also be contributing to a detrimental sleep onset association.

### Reliability of the Coding System

Overall inter-rater percentage agreement the final version of the coding system was 74% which is above the guideline threshold of 70% (28). 100% agreement was achieved for the categories “time domains” and “child global status,” in line with Lesser’s 2019 study, which found percentage agreement between two observers for sleep onset, sleep offset, and night wakings to be 100%. “Location” and “activity” had a lower but acceptable percentage agreement (80% and 74% respectively), though on most occasions when the two coders “disagreed” in these categories it was because the timestamps of their observations were more than 5 s apart. There was 0% agreement for “physical interaction” due to the coders disagreeing on the start and end time of the behavior, since the view of the parent and child was partially obstructed by bedding. Agreement for “physical environment” could not be assessed using the final coding system because there were no changes in the physical environment during the segment of video.

### Accuracy of Parental Report

Parental interview was compared with objective coded data from the overnight video study relating to timing of sleep and number of night wakings (Table 4). Sleep schedule (sleep onset and sleep offset time) was reasonably accurately reported, but the number and duration of night wakings (determinants of the child’s sleep quality) were consistently under-reported. This was generally because the child self-soothed quietly without signaling for the parental attention.

**Table 4:** Parent report vs. objective measurement of child's sleep schedule and sleep quality. Sleep schedule was accurately reported by parents; however, report of child's sleep quality was poorly reported. All parents under-reported the frequency and duration of night wakings.

	Variable	Participant 1		Participant 2		Participant 3	
		Reported	Observed	Reported	Observed	Reported	Observed
**Sleep schedule**	Sleep onset time	20:30	20:37	20:30	20:15	21:30	21:04
	Sleep offset time	06:30	06:10	07:00	06:15	06:10	06:10
**Sleep quality**	Number of night wakings	4	12	0	2	0	1
	Total duration of night wakings (min)	12.0	26.0	0	1.8	0	3.1

Additionally, we noted that parents tended to omit information about the child’s bedtime routine and overnight behavior. For example, when asked about the physical environment of the child’s bedroom, one parent described it as quiet and with dim lighting. However, on observation, the main bedroom light was turned on and off several times, family members walked in and out of the room, and a mobile phone in the room started ringing loudly during the settling process. The child was visibly distracted by these events which disrupted the otherwise soothing atmosphere in the bedroom.

There were examples of a mismatch between parent report and HVSG observation in all three cases, particularly in regards to parent or child behavior. A parent whose child did not have CI reported that the child signaled whenever he woke up in the night and that she had to be present in the room in order for him to fall asleep. However, following coding of the child’s overnight footage, it was observed that the child was able to self-soothe on multiple occasions following night waking without the presence of the parent.

For all participants, a graphical visual summary of the bedtime and overnight activity was generated using BORIS^©^ (**Supplementary Figures 1** and **2**).

## Discussion

CI is the most common sleep disorder experienced by children, yet diagnosis is guided by subjective parental report. Despite parent-child interactions at bedtime being key to the etiology of CI, there is no accepted method to assess these interactions objectively. This study aimed to develop a new tool to objectively assess overnight interactions between children and their parents, which could assist in the identification of behaviors contributing to recurring CI. We have created a reliable and reproducible coding system, the Overnight Parent-Child Interaction Coding (OPIC) System, to fulfill this purpose.

The OPIC system demonstrated acceptable reliability. This is particularly significant considering there were 26 behaviors accounted for in the final version of the coding system. The poor percentage agreement for two of the coding categories (“physical environment” and “physical interaction”) can be partially explained by the infrequent use of these codes in the segment of video used to test reliability. Reliability should be tested again using different footage samples. Nevertheless, the reliability of the coding system is a promising starting point for future development.

We have shown that this coding system can be applied to three separate overnight videos involving complex, lengthy bedtime routines, and nighttime interactions. The coding system has been used to support parental interview as an investigative tool both in children with CI and without CI and yields additional data to parental report. Applying the coding system to these videos has exposed aspects of the bedtime routine, such as changes in the physical environment, which parents did not disclose during the interview. For example, family members entering and leaving the room and the main light being turned on and off. The parents may have omitted these details from the interview due to recall error, though the clinical interview was undertaken the following day to minimize this risk. It is also possible that parents were selective in their disclosure, either due to an awareness that the behavioral dynamic was inappropriate or because they did not believe it to be relevant for the interviewer. The significant mismatch between one parental account of an idealized settling environment, despite the observed reality which was noisy and at times chaotic is interesting and may reflect social desirability bias (11).

The comparison between parental report and observation of the child’s sleep schedule and sleep quality in this small sample supports previous reports that parents are poor historians in terms of their child’s sleep quality (8, 9). This finding is significant because problems with *maintaining* sleep are of equal importance as *initiating* sleep in the diagnostic criteria for CI. Therefore, if parents are unable to accurately report their child’s ability to maintain sleep, an alternative investigation to parental interview to support a diagnosis is warranted.

In practice, HVSG and coding of parent-child interactions could be used to aid clinical management. CI is treated using behavioral intervention, with the clinician providing advice based on reported child and parent behaviors (30). This usually involves removing problematic sleep onset associations and advice on sleep hygiene (31). However, it seems likely that advice given by the clinician could be improved if the clinician had objective evidence of the nature of parent-child interactions during the bedtime routine. This could facilitate a more targeted family-centered management plan. This plan could be enhanced by focusing on specific examples of problematic and/or constructive parent-child interactions, which could be replayed from HVSG footage. Video feedback has been used in psychological interventions to effectively guide parents with child behavior management (32–35) and has been shown to improve child attachment security (34), parent mental health status (32), and parent self efficacy (36) [confidence in their own ability to succeed (37)] in comparison to simple verbal feedback.

Following behavior coding using BORIS^©^, the software was used to create a graphical output of all codes recorded during an observation. This displayed all activity which occurred in the bedroom, including child and parent activity and changes in physical environment. This graph could also provide a visual educational tool for the families of children with CI to demonstrate a complex and excessively long bedtime routine, compared to an appropriate graphical representation of a brief but soothing bedtime routine (see **Supplementary Figures 1** and **2**).

While the OPIC system demonstrated acceptable reliability, further development and testing is warranted as the current version has some limitations. Firstly, coding was time-consuming (the viewing of segments, at times, required up to four times the duration of the whole video to enable scoring where significant interactions between the parent and child took place). This limits its application in clinical practice. To overcome this limitation the OPIC system could be further simplified to focus on core behaviors of interest. Alternatively, the clinician could code shorter segments of the video rather than the entire bedtime routine. For example, 1 min for every 5 min of video. This would take less time than coding the whole bedtime routine and would still enable video feedback, including examples of undesirable interactions.

Another limitation of this study was the approach to IRR testing. Only two coders tested the coding system, both of whom were involved in its development, and were therefore familiar with the coding system. To develop the coding system further and enable its validation as an investigative tool, it should undergo testing by a larger group of independent coders.

In the future, HVSG combined with objective coding systems based on OPIC could be a promising supplement to actigraphy as an objective assessment of children’s sleep. HVSG could also offer advantages over actigraphy for a sub-sample of children with sensory processing difficulties, such as those with autism spectrum disorder, who might not tolerate a wrist-worn device (38).

A systematic approach to quantifying child and parent behavior in CI would offer an objective outcome measurement tool for CI interventions. The effectiveness of behavioral interventions in childhood CI has only been tested previously using sleep diaries (39), questionnaires (40–42), and actigraphy (39, 40, 42). Robust objective assessment of behavioral interventions in childhood CI could provide a more reliable insight into the outcome of these interventions, since researchers could directly observe the extent to which the management plan has been implemented by parents.

To conclude, the OPIC system, when applied to home videosomnography, offers the potential for personalized interventions in the clinical management of behavioral insomnia and an objective outcome measurement in clinical research. The paradigm of sleep scoring and video observation is core to mainstream sleep diagnostics and the OPIC system applies similar approaches to a behavioral setting. Software could be adapted within conventional commercial sleep systems to accommodate such an approach.

## Data Availability Statement

The raw coding data supporting the conclusions of this manuscript will be made available by the authors, without undue reservation, to any qualified researcher. The video data for this manuscript are not publicly available because this would breach the terms of our ethical approval for this study. Requests to access the datasets should be directed to Dr Catherine Hill, c.m.hill@soton.ac.uk. 

## Ethics Statement

The studies involving human participants were reviewed and approved by Health Research Authority and South Central – Hampshire A Research Ethics Committee (reference 18/SC/0012) University Hospital Southampton NHS Foundation Trust (reference CHI0913). Written informed consent to participate in this study was provided by the participants’ legal guardian/next of kin.

## Author Contributions

The study was designed by LG, KB, and CH. Recruitment and data collection was completed by LG. Iterative development of the coding system and the coding of video to calculate inter-rater reliability were done by LG and CH. Data analysis was completed by LG.

## Funding

This study was funded by the University of Southampton.

## Conflict of Interest

The authors declare that the research was conducted in the absence of any commercial or financial relationships that could be construed as a potential conflict of interest.
