# Supplementary material for: Video Analysis of Parent–Child Interactions in Behavioral Sleep Disorders: Development of a Scoring Algorithm
Source: Front Psychiatry. 2019 Nov 22;10:861. doi: 10.3389/fpsyt.2019.00861 (PMC6882929; doi:10.3389/fpsyt.2019.00861)
Supplement: Supplementary file 1 [file DataSheet_1.docx]

 Supplementary Material

Supplementary Figure 1. Graphical output from BORIS© demonstrating all overnight activity in a child with chronic insomnia. Diagram shows the overnight behaviour of the child, mother and grandmother. The grandmother was responsible for the bedtime routine and putting the child to bed – she was present at sleep onset. The bedtime routine was prolonged and involved stimulating parent-child interactions and changes in sound/light levels. There was over an hour between the start of the bedtime routine and the child’s sleep onset. The child co-slept with his mother, who joined after midnight. There were twelve night wakings, following several of which the child signalled for the grandmother or mother.

**Supplementary Figure 2.** **Graphical output from BORIS© demonstrating all overnight activity in a child without chronic insomnia.** Diagram shows the overnight behaviour of the child and mother. The mother read to the child during the bedtime routine, which lasted just over 30 minutes. The mother remained in the room (sitting silently on the floor) for sleep onset. However, the child woke once in the night (at 02:00) and managed to self soothe, therefore his mother’s presence in the room is not a sleep onset association.
